# Relationship between mobility, violence and major depression among female sex workers: a cross-sectional study in southern India

**DOI:** 10.1136/bmjopen-2016-011439

**Published:** 2016-09-09

**Authors:** Sangram Kishor Patel, Deepika Ganju, Parimi Prabhakar, Rajatashuvra Adhikary

**Affiliations:** 1 HIV and AIDS Program, Population Council, New Delhi, India; 2 India HIV/AIDS Alliance, Sarovar Center, Hyderabad, Telangana, India

**Keywords:** EPIDEMIOLOGY, MENTAL HEALTH

## Abstract

**Background:**

The relationship between mobility, violence and mental health has largely been unexplored in developing countries. This study screens for signs of major depression, and assesses its association with mobility and violence among female sex workers (FSWs) in southern India.

**Methods:**

Data (N=2400) for this study were used from a cross-sectional Behavioral Tracking Survey (BTS-2014) conducted among FSWs from a southern state of India as part of the Avahan programme. Major depression of FSWs was assessed using the Patient Health Questionnaire-2 depression scale. Descriptive statistics, frequency, bivariate, interaction effect and multivariate logistic regression techniques were used for the analysis.

**Results:**

More than one-fourth of FSWs (29%) screened positive for major depression. The likelihood of screening positive for major depression was 6 times higher among FSWs who were both mobile for sex work outside their district of residence and had experienced any violence (combined association) during the past 1 year (62% vs 19%, adjusted OR 6.1, 95% CI 4.4 to 8.6) compared with those who reported neither. The individual association results show that FSWs who reported being mobile outside the district, and FSWs who were beaten or raped in the past 1 year, were 3 times more likely to screen positive for major depression.

**Conclusions:**

The findings indicate that violence and mobility are independently associated with major depression among FSWs. The combined association of mobility and violence poses a greater risk to the mental health of FSWs than their independent association. These results point to the need for creating an enabling environment for FSWs to enhance existing efforts to reduce the spread of HIV and mental health problems. The study highlights that HIV prevention efforts among FSWs in India require evidence-based research and integrated programme approaches to address mental health issues.

**Strengths and limitations of this study**

- This is one of the studies to understand the individual and combined association of mobility and violence on the mental health of female sex workers (FSWs), a high-risk population in India. Both the individual and combined association of mobility and violence place FSWs at high risk of major depression.
- The study findings would add to the body of research on violence, mental health and HIV vulnerability among FSWs, and provide valuable inputs to inform HIV prevention and mental health programmes in India and elsewhere.
- The variables considered in this study are based on self-reported responses (eg, violence), and may have been under-reported perhaps due to the stigma associated with reporting violence or FSWs' perception of violence based on only the severity of the experience and the reference period of the violence occurred.
- The findings of this study, which are based on a selected sample of FSWs—those who are members of community-based organisations, cannot be generalised to all FSWs across India. However, the study results can be generalised to other geographical areas with similar sex work characteristics.

## Introduction

Mental health issues have been an integral part of the prime agenda of many countries but have been neglected in the developing world. The Mental Health Atlas 2011 revealed that mental health-related disorders contribute to nearly 13% of the total burden of disease globally.^1^ Mental health is becoming a serious problem in developing countries. Recent data from developing countries show that more than 75% of people who suffer from mental disorders do not receive any treatment.^1^ Studies also reveal that depressive symptoms among women are associated with experiences of violence, including intimate partner violence.^2^ The WHO^3^ also confirms that violence against women is one of the leading causes of depression among women. Evidence shows that female sex workers (FSWs), a high-risk population group, are highly vulnerable to different forms of violence,^4–7^ and violence perpetrated against FSWs is increasingly associated with different mental health outcomes.^6–13^ Further, FSWs who are mobile have higher chances of reporting depressive symptoms^14^ ^15^ because of their increased likelihood of engaging in high-risk behaviours and experiencing violence compared with their non-mobile counterparts.^4^ ^16–20^

Globally, mental health problems and the risks that sex workers face have been empirically documented. These studies report a high correlation between mental distress and loneliness, isolation, fear;^21^ anxiety;^22^ post-traumatic stress disorder (PTSD);^14^ ^23^ risky sexual behaviour;^23^ ^24^ drug and alcohol abuse;^25^ and violence^24^ ^26^ among sex workers. Given the worldwide concern regarding the spread of HIV/AIDS through sex work/commercial sex, most preventive measures have focused on the risks associated with the transmission of HIV/AIDS rather than on other health issues, including mental health.^2^ ^24^ As a result, the psychosocial problems and mental health needs of sex workers have been largely ignored. Although the literature documents the relationship between mobility, violence and HIV vulnerability among FSWs,^4^ ^6^ ^16^ ^17^ ^27–29^ there is a lack of research focusing on the relationship between mobility and violence, and mental health issues, in developing countries. While it has potential policy and programmatic implications, the mental health issues of FSWs have been largely understudied. To address this knowledge gap, this study explores the mental health issues of FSWs. It specifically aims to screen for signs of major depression, and to assess the independent and combined associations of mobility and violence with major depression among FSWs in southern India. The study findings would add to the body of research on violence, mental health and HIV vulnerability among FSWs, and provide valuable inputs to inform the HIV prevention programme in India and elsewhere.

## Data and methods

This study is referred (particularly the sections on research setting and design, sampling and sample size, and ethics statement) from the data and methods section of our previously published study^30^ which is also based on the same survey data set and methodology.

### Research setting and design

Data on FSWs (N=2400) were used from the Behavioral Tracking Survey—2014 (BTS) for this study. This cross-sectional survey was conducted among FSWs, registered members of community-based organisations (CBOs) during April to May 2014 in Andhra Pradesh, India. The survey monitors critical components of the Avahan programme (Avahan-India's AIDS Initiative), including community mobilisation, condom use, sexually transmitted infection (STI) management, behaviour change communication, sustainability and advocacy along with social entitlements and financial inclusion among FSWs.

Avahan programme, a large-scale HIV prevention programme, was launched in 2003 with key populations across six high-HIV prevalence states in India. One of its goals was to mobilise communities of FSWs and high-risk men who have sex with men (HR-MSM)/transgenders (TGs) to manage and implement prevention programmes themselves.^31^ India HIV/AIDS Alliance was instrumental in strengthening the CBO activities towards community mobilisation initiative in Andhra Pradesh. Avahan's Common Minimum Program (CMP) for community mobilisation among populations at risk of HIV infection (FSWs, HR-MSM and TGs) was introduced during mid-2012 to focus on: crisis response systems, financial inclusion savings and loans, access to social entitlements, advocacy for HIV services and organisational development—governance, leadership, project management, resource mobilisation.^32^ CMP works closely with key populations to help them build CBOs that can advocate for the basic needs of their members through community-based groups (CBGs). CBGs are empowered and have an understanding about shared responsibility towards increased access and usage of project services. In each community mobilisation implemented district, CBOs at the block level are further divided into two organisations: (1) site level associations (SLAs—at the mandal/cluster/ward level), and (2) CBGs as the grassroots/primary organisations. Usually one CBO has been formed for ∼2000–3000 registered FSWs. Each SLA is monitored by a CBG facilitator (one CBG facilitator for ∼200–250 registered FSWs). One CBG has ∼10–15 registered FSWs.^32^

### Sampling and sample size

Six districts (Ananthapur, Chittoor, Karimnagar, Khammam, Nalgonda and Warangal) were purposively selected in the BTS-2014, where the community is being strengthened through CBO activities under the Avahan programme. In each selected district, a sample of 400 FSWs was covered in the survey. The sample size was calculated based on a number of assumptions, including prevalence of consistent condom use (CCU; 50%), expected change in CCU over the next few years (15 percentage points), confidence level (95%), power of estimate (90%) and design effect (1.7). The sampling frame was prepared by considering the number of FSWs registered in each CBG under different CBOs. A two-stage cluster random sampling method was used to select respondents within the CBOs. In the first stage, the required numbers of CBGs within different clusters/wards were selected based on probability proportional to size. In the second stage, the required numbers of FSWs were randomly selected for interview within each selected CBG. A total of 2400 FSWs were interviewed in the BTS-2014. All interviews were conducted by trained female investigators with verbal and written skills in Telugu, the local language of Andhra Pradesh. The survey instrument (structured questionnaire) was developed in English and translated into Telugu language. The questionnaire was pretested in communities similar to the survey sites. All the interviews were held in a private location specifically hired for the survey or in a location convenient to the study participants. Field staff checked the data immediately after the interviews to ensure accuracy and completeness of the filled-in questionnaires. A user written computer program in CSPro (V.5.0) was used for double data entry by trained data entry officers.

### Ethics statement

Verbal consent was obtained from all respondents prior to participation in the interview, and steps were taken to ensure confidentiality. On receipt of verbal consent from the eligible respondent, the interviewer signed the oral consent form on behalf of the respondent. In this survey, oral consent was recommended as information on the respondent's background and behavioural characteristics were collected and no blood or other specimen collection was carried out. In this survey, women aged 18 years or more who had sex in exchange of cash/kind in the past 1 month were considered to be FSWs and the information was collected accordingly. No names and addresses were recorded on the questionnaires. No compensations were provided to the participants for their time in the study, but were referred to local project services undertaken by implementing agencies in the study districts.

### Measures

Based on the literature review and availability of the variables in the questionnaire, different variables were considered for this study. The sociodemographic variables considered in the analysis were age (<30, ≥30 years); marital status (never married, currently married and formerly married (widowed/divorced/separated/deserted)); formal education (no, yes); primary place of solicitation for sex work (home, public places/street, brothel/lodge, mobile phones); duration of sex work (<5, ≥5 years); under debt at the time of survey (no, yes); inconsistent condom use with clients (occasional/regular; no, yes) and self-reported STI symptoms in past 1 year (no, yes) were also assessed. Other variables considered were mobility for sex work in the past 2 years (mobility among FSWs can be defined as those who were mobile for sex work at least once in past 2 years, either within or outside the district from current place of residence; no mobility, mobility within the district and mobility outside the district); experience of violence in the past 1 year (no, yes; violence: any physical or sexual violence as reported by FSWs in the past 1 year) and perpetrators of violence against FSWs (clients, husband/partners, police, brokers/pimps/goons and strangers/co-workers/others).

### Major mental depression

In this study, mental health status constitutes the dependent variable and was measured using the Patient Health Questionnaire-2 (PHQ-2) depression scale. The PHQ-2 scale is used for screening mental depression based on the frequency of depressed moods and anhedonia (ie, inability to experience pleasure from activities usually found enjoyable).^33^ FSWs were asked two questions (from the PHQ-2 scale) during the survey: over the past 2 weeks, how often have you been bothered by any of the following problems: (1) little interest or pleasure in doing things; (2) feeling down, depressed or hopeless. Answers were recorded in one of the following four categories, 0: not at all; 1: several days; 2: more than half the days and 3: nearly every day. The total score ranged from 0 to 6, and in the present study, we have considered a previously validated score of 3 on the PHQ-2 scale as the optimal cut-off point to indicate that the screen was positive for depression (1=having major depression (≥3 score) and 0=no and low depression (<3 score)). Guidelines for cut-off scores for major depression are presented elsewhere.^33^

### Data analysis

Means, SDs, proportions and bivariate analyses were used to describe the association of sociodemographic characteristics, violence, mobility and major depression among FSWs. To test the significance, the respective p values for the bivariate analysis were calculated through χ^2^ test. Adjusted ORs (AORs) and their 95% CIs were estimated through multivariate logistic regression, adjusting for sociodemographic characteristics, to assess the relationships of the degree of mental depression with the potential independent variables. The mobility and violence interaction effects were also used to assess the association of the combined effect of these two variables with the outcome indicator (eg, major depression). The interaction effect used here can be defined as ‘the differing effect of one independent variable (mobility) on the dependent variable (major depression), depending on the particular level of another independent variable (violence)’.^34^ This is a useful and common concept in social and health science research. All analyses were conducted using STATA software (V.11.2).

## Results

The mean age of the study participants was 31 years (SD=5.8 years). More than half of the FSWs in the study did not have any formal education (56%), some were currently married (67%), some were using their mobile phone as their primary mode of solicitation for sex work (54%) and some had been practising sex work for <5 years (54%; table 1). Two-thirds of FSWs were in debt at the time of survey. Of the 2400 FSWs included in the analyses, nearly one-fifth (19%) had travelled to places outside the district and one-third (33%) had travelled to places within the district from their current place of residence for sex work. There are significant differences between the groups by mobility for sex work (non-mobile, within district and outside district) by background characteristics. Nearly one-fourth (24%) had experienced any violence in the past 1 year. Experience of violence was significantly higher among FSWs who were mobile outside the district, followed by those who were mobile within the district. Similarly, mobility for sex work outside the district was significantly higher among FSWs who had practiced sex work for more than 5 years, were in debt, had experienced violence and had experienced any STI symptoms. In addition, mobility outside the district of residence was higher among FSWs who were over 30 years, used a mobile phone for sex work solicitation and were currently married or formerly married.

**Table 1:** Background characteristics of female sex workers by mobility in Andhra Pradesh, India, 2014

Background characteristics	Per cent (n) or mean (SD)	Mobility for the sex work			p Value
		No mobility	Within district	Outside district	
Age (mean, SD)	30.9 (5.8)	30.7 (6.0)	31.2 (5.7)	31.0 (6.0)	
Age (years)					0.100
<30	43.3 (1040)	45.5	40.9	41.9	
≥30	56.7 (1360)	54.5	59.1	58.0	
Formal education					0.000
No	56.2 (1350)	60.3	56.4	45.5	
Yes	43.8 (1050)	39.7	43.6	54.5	
Marital status					0.000
Never married	5.0 (119)	6.6	3.3	3.8	
Currently married	66.5 (1596)	68.4	67.0	60.7	
Formerly married	28.5 (685)	25.0	29.7	35.5	
Primary place of solicitation for sex work					0.000
Home-based	13.9 (333)	19.9	8.9	7.0	
Public places/street	4.5 (109)	4.3	4.8	4.6	
Brothel and lodge-based	27.6 (663)	26.9	30.7	24.0	
Mobile phones	54.0 (1295)	48.9	55.5	64.2	
Duration of sex work (years)					0.005
<5	54.3 (1304)	56.6	54.8	47.7	
≥5	45.7 (1096)	43.4	45.2	52.3	
Under debt at the time of survey					0.013
No	33.9 (814)	35.5	35.0	28.0	
Yes	66.1 (1586)	64.5	65.0	72.0	
Mobility for sex work in past 2 years					
No mobility	48.3 (1162)	–	–	–	
Mobility inside district	32.7 (785)	–	–	–	
Mobility outside district	19.0 (453)	–	–	–	
Inconsistent condom use with clients (occasional/regular)					0.001
No	71.2 (1709)	67.7	74.5	74.4	
Yes	28.8 (691)	32.3	25.5	25.6	
Self-reported STI symptoms in past 1 year					0.000
No	64.5 (1549)	76.8	57.4	45.5	
Yes	35.5 (851)	23.2	42.6	54.5	
Experience of violence in past 1 year					0.000
No	76.0 (1824)	85.7	75.0	52.8	
Yes	24.0 (576)	14.3	25.0	47.2	
Total sample	100.0 (2400)	100.0 (1162)	100.0 (785)	100.0 (453)	

SD stands for SD.

p Value were calculated through the χ^2^ test.

STI, sexually transmitted infection.

More than one-fourth of FSWs (29%) were screened positive for major depression. Just under one-fourth had reported being depressed for more than half the day to every day (table 2). Nearly one-third of FSWs who were mobile outside the district reported being depressed for more than half the day to every day compared with 22% and 18%, respectively, who were mobile inside the district and were not mobile (table 2). The AORs in table 3 were adjusted for age, education, marital status, primary place of sex work solicitation and duration of sex work. Results indicate that experience of violence and mobility for sex work were independently associated with major depressive symptoms. The odds of screening positive for major depression were three times higher among FSWs who reported being beaten or raped at least once in the past 1 year than others (50% vs 23%, AOR 3.0, 95% CI 2.4 to 3.6). Similarly, the chances of having major depressive symptoms were three times higher among FSWs who were mobile outside the district (48% vs 22%, AOR 2.8, 95% CI 2.2 to 3.6) and 40% higher among those who were mobile within the district of residence (30% vs 23%, AOR 1.4, 95% CI 1.2 to 1.8) compared with those who were not mobile for sex work. Further, FSWs who were both mobile outside the district and reported violence were six times more likely to be at risk for major depressive symptoms (62% vs 19%, AOR 6.1, 95% CI 4.4 to 8.6) than FSWs who were not mobile and did not experience violence. Similarly, FSWs who were both mobile within the district and reported violence were three times more likely to be at risk for major depressive symptoms (43% vs 19%, AOR 3.0, 95% CI 2.2 to 4.2) than FSWs who were not mobile and did not experience violence. The odds of screening positive for major depression were seven times higher among FSWs who reported any violence perpetuated by the police (73% vs 23%, AOR 7.4, 95% CI 4.5 to 13.0), and five times higher among FSWs reporting violence perpetuated by a broker, pimp or goons (62% vs 23%, AOR 5.0, 95% CI 3.4 to 7.6) compared with those who had not experienced any violence (table 4).

**Table 2:** Mental health status as reported by female sex workers in Andhra Pradesh, India, 2014

Indicators	Per cent	Mobility status			p Value
Over the past 2 weeks, how often have you been bothered by any of the following problems		No mobility	Within district	Outside district	
Little interest or pleasure in doing things					0.000
Not at all	44.1	44.2	47.1	38.4	
Several days	33.7	38.7	29.7	27.8	
More than half the days	19.9	15.4	20.4	30.7	
Nearly every day	2.3	1.6	2.8	3.1	
Feeling down, depressed or hopeless	** **	** **	** **	** **	0.000
Not at all	54.7	62.1	52.2	39.7	
Several days	23.4	19.6	25.6	29.4	
More than half the days	16.9	13.9	18.0	22.7	
Nearly every day	5.0	4.3	4.2	8.2	
Mental health screening status					0.000
No/low depression	70.7	78.3	69.9	52.5	
Major depression	29.3	21.7	30.1	47.5	
N	100.0 (2400)	100.0 (1162)	100.0 (785)	100.0 (453)	

The mental health status index was calculated considering a score of 3 on the PHQ-2 scale as the optimal cut-off point to indicate that the screen was positive for depression (1=having major depression (≥3 score) and 0=no and low depression (<3 score)).

PHQ-2, Patient Health Questionnaire-2.

**Table 3:** Association between violence, mobility and major mental depression among female sex workers, Andhra Pradesh, India, 2014

Characteristics	Screening of major depression	AORs (95% CIs)
Mobility for sex work in past 2 years		
No mobility	21.7 (252)	Reference
Mobile inside district	30.0 (236)	1.4 (1.2 to 1.8)***
Mobile outside district	47.5 (215)	2.8 (2.2 to 3.6)***
Experience of violence in past 1 year		
No	22.9 (418)	Reference
Yes	49.5 (285)	3.0 (2.4 to 3.6)***
Mobility and experience of violence		
No mobility and not experienced violence	18.5 (184)	Reference
Mobility inside district but not experienced violence	25.6 (151)	1.4 (1.1 to 1.8)***
Mobility outside district but not experienced violence	34.7 (83)	2.1 (1.5 to 2.9)***
No mobility but experienced violence	41.0 (68)	2.8 (1.9 to 3.9)***
Mobility inside district and experienced violence	43.4 (85)	3.0 (2.2 to 4.2)***
Mobility outside district and experienced violence	61.7 (132)	6.1 (4.4 to 8.6)***
N=2400	29.3 (703)	

***, ** and * indicate values are significant at 1%, 5% and 10% level of significance respectively. AORs are adjusted for age, education, marital status, primary place of sex solicitation, duration of sex work.

AORs, adjusted ORs.

**Table 4:** Association between perpetrators of violence against the FSWs and major depression among FSWs, Andhra Pradesh, India, 2014

Perpetrators of violence against the FSWs	Any violence	Screening of major depression	AORs (95% CIs)
No violence	76.0 (1824)	23.0 (418)	Reference
By clients	8.2 (196)	44.4 (87)	2.4 (1.8 to 3.2)***
By husband/partners	6.0 (145)	40.7 (59)	2.2 (1.5 to 3.1)***
By police	2.6 (63)	73.0 (46)	7.4 (4.5 to 13.0)***
By brokers/pimps/goons	4.6 (111)	62.2 (69)	5.0 (3.4 to 7.6)***
By strangers/co-workers/others	2.5 (61)	39.3 (24)	2.0 (1.2 to 3.4)**
N=2400	100.0 (2400)	29.3 (703)	

*** and ** indicate values are significant at 1% and 5% level of significance respectively. AORs are adjusted for age, education, marital status, primary place of sex solicitation, duration of sex work.

AORs, adjusted ORs; FSW, female sex worker.

## Discussion

Findings from the study show that more than half of the FSWs were mobile for sex work either outside the district or within the district of residence, and nearly one-fourth reported experiencing violence in Andhra Pradesh. This study also illustrates that screening positive for major depression is higher among mobile FSWs than others. The study findings confirm that mobility and experience of violence are significantly associated with reporting major depressive symptoms in southern India. According to previous research, mobility for sex work may not be directly associated with depressive symptoms, but mobility can escalate FSWs' risk of being exploited, experiencing violence, and forced and unprotected sex as a result of operating in new environments and lack of social support,^28^ ^35^ which leads to reproductive and mental health problems.^15^ ^24^ Further, findings from other studies explain that the main reasons for FSWs' mobility are to increase their client base, enhance their earnings, and to escape stigma and discrimination^16^ ^36^ and police harassment.^37^

Findings from various countries document that during the course of mobility, FSWs have higher chances of encountering different vulnerabilities including violence.^4^ ^6^ ^28^ Similar findings are observed in this study, which shows that nearly one-fourth of FSWs who visited places outside their district for sex work reported any violence; similar levels of violence are reported in studies from other Asian countries.^11^ ^38^ ^39^ Our study further highlights that mobile sex workers mainly face violence perpetuated by clients, the police, sexual partners or brokers/pimps/goons. The study adds further that FSWs who had experienced violence were more likely to report major depressive symptoms than others. Previous studies support this finding that FSWs who are sexually and/or physically abused are at higher risk of depression and PTSD along with STI/HIV.^2^ ^7^ ^13–15^ ^23^ Results from this study indicate that if the police perpetuated violence against FSWs, the likelihood of reporting major depression increased seven times, followed by broker/pimps/goons by five times. These results suggest the need for more programmatic attention to protect FSWs who are an extremely marginalised population. While the effect of mobility and violence on reproductive and sexual health outcomes among sex workers is well established,^4^ ^6^ ^17^ ^29^ this study has gone a step further and established both the individual and combined association of mobility and violence with major depression among FSWs. Findings of the study reveal that FSWs who are mobile outside their usual district of residence and have experienced violence are six times more likely to screen positive for major depression than those who reported neither. These multiple vulnerabilities of FSWs are a serious concern, and require urgent programmatic interventions to build an enabling environment for sex workers.

While this study underlines the strong association between violence, mobility and major depression among FSWs, the findings should be interpreted with caution in light of certain limitations. First, the main variables considered in this study were based on self-reported responses, and the limitations of self-reported data are widely recognised.^40^ For example, violence may have been under-reported perhaps due to the stigma associated with reporting violence or sex workers' perception of violence based on only the severity of the experience and reference period when the violence occurred. This study is also based on the assumption that FSWs who reported experiencing violence and mobility in the recent past may have also experienced similar vulnerabilities since their entry into sex work. Second, the inclusion of other variables, such as psychological violence, treatment-seeking behaviour and the availability of mental health services, could have provided greater insights into the mental health situation of FSWs, and provided additional input for the design of future policies and programmes. Third, as FSWs in this study are part of the Avahan programme, they may not be representative of all FSW populations as they are part of a programme aimed at community engagement and empowerment, and therefore, the levels of violence and the proportion that have screened positive for major depression may be lower than in other sex worker populations. Fourth, the findings of this study, which are based on a selected sample of FSWs who were members of CBOs, cannot be generalised to all FSWs across India. However, the study results can be generalised to other geographical areas with similar sex work characteristics. Nonetheless, these limitations do not compromise the internal validity of the data: our findings are consistent with the results of previous studies that have assessed the association between violence, mobility, sexual risk behaviours and mental health problems.

To conclude, the study shows that violence and major depression are high in this marginalised and high-risk group. This study adds further that the individual and combined association of mobility and violence place FSWs at greater risk of major depression. The study findings underscore the need to strengthen the existing HIV prevention programmes for FSWs by incorporating strategies to address mental health issues. So far, the Indian government's National AIDS Control Program guidelines as well as Avahan programme in India have ignored the mental health problems of key populations, including FSWs. More focused efforts are needed to create an enabling environment and crisis response services both at sex workers' place of origin and place of destination. Further, it would be beneficial to identify high-risk zones of mobility and violence through geographical mapping, build awareness about legal rights, promote self-defence mechanisms and initiate more interaction between CBOs, civil society and law enforcement agencies to reduce vulnerabilities, including mental health problems, among FSWs (with a special focus on high mobility zones). Governments and agencies working with FSWs should highlight the barriers in addressing mental health, initiate counselling services, and provide information on mental health issues and associated risk factors. Additionally, they should advocate for including mental health services at voluntary counselling and testing centres, along with other public and private health facilities, through an integrated services approach. Campaigning for additional resources for mental health services should also be given priority along with economic strengthening of FSWs. More importantly, the government and policymakers need to acknowledge that mental health is an important issue among FSWs and other marginalised populations. Future research could provide critical information on several key issues that would have implications for HIV and mental health policies and programmes. Studies could explore the extent to which FSWs' degree of mobility and exposure to different kinds of violence is associated with reproductive health and mental health problems. This study suggests further research and advocacy to ensure that mental health issues are appropriately addressed for marginalised populations.
